# Magnesium intake and colorectal cancer risk in the Netherlands Cohort Study

**DOI:** 10.1038/sj.bjc.6603577

**Published:** 2007-02-06

**Authors:** P A van den Brandt, K M Smits, R A Goldbohm, M P Weijenberg

**Affiliations:** 1 Department of Epidemiology, Maastricht University, PO Box 616, 6200 MD Maastricht, The Netherlands; 2 TNO Nutrition and Food Research, PO Box 360, 3700 AJ Zeist, The Netherlands

**Keywords:** colorectal cancer, cohort studies, magnesium, BMI

## Abstract

Energy-adjusted magnesium intake was nonsignificantly inversely related to risk of colorectal cancer (*n*=2328) in the Netherlands Cohort Study on Diet and Cancer that started in 1986 (*n*=58 279 men and 62 573 women). Statistically significant inverse trends in risk were observed in overweight subjects for colon and proximal colon cancer across increasing quintiles of magnesium uptake (*P*-trend, 0.05 and 0.02, respectively). Although an overall protective effect was not afforded, our results suggest an effect of magnesium in overweight subjects, possibly through decreasing insulin resistance.

Two recent prospective cohort studies among women showed an inverse association between magnesium intake and colorectal cancer (Larsson *et al*, 2005) and colon cancer only (Folsom and Hong, 2006). Magnesium supplementation reduced colon cancer risk in animal experiments (Mori *et al*, 1993). Magnesium plays a role in genomic stability and DNA repair (Hartwig, 2001; Larsson *et al*, 2005) and may reduce hyperinsulinaemia (Paolisso *et al*, 1992; Rodriguez-Moran and Guerrero-Romero, 2003), a risk factor for colorectal cancer (Giovannucci, 1995; Schoen *et al*, 1999). In the Netherlands Cohort Study (NLCS), we investigated colorectal cancer in both sexes in relation to magnesium intake, particularly in overweight subjects, given the suggested beneficial effects of magnesium on insulin resistance (Fung *et al*, 2003).

## MATERIALS AND METHODS

The NLCS started in 1986 and included 58 279 men and 62 573 women aged 55–69 years. At baseline, cohort members completed a mailed, self-administered questionnaire on dietary habits, anthropometry, and other risk factors for cancer (Van den Brandt *et al*, 1990a). Habitual consumption of food and beverages during the year preceding baseline was assessed using a 150-item semiquantitative food frequency questionnaire (Goldbohm *et al*, 1994). From this, nutrient intakes were calculated from the 150 food items using the computerized Dutch food composition table (Nevo-table, 1986). Nutrient intake was adjusted for energy intake by the residual method (Willett and Stampfer, 1986).

Data were processed and analysed using the case–cohort approach, enumerating the cases for the entire cohort, and estimating the person-years at risk from a subcohort of 5000 subjects, which was randomly sampled from the entire cohort immediately after the baseline measurement and followed up for vital status. Follow-up for cancer incidence is established by record linkage with the Netherlands Cancer Registry and PALGA, a nationwide pathology database (Van den Brandt *et al*, 1990b). After 13.3 years of follow-up, a total of 2679 incident colorectal cancer cases were reported. Cases and subcohort members were excluded if they reported cancer other than non-melanoma skin cancer, or had incomplete data for diet, anthropometry, or confounders. Finally, 4125 subcohort members and 2328 colorectal cancer cases were available for analysis.

## STATISTICAL ANALYSIS

Incidence rate ratios (RR) and 95% confidence intervals for colorectal cancer and subsites were estimated using Cox proportional hazards models (Cox, 1972), with Stata software (Cleves *et al*, 2002). Standard errors were estimated using the robust Huber–White sandwich estimator to account for additional variance introduced by sampling from the cohort (Schoenfeld, 1982). All RRs are adjusted for confounders that contributed significantly to the model or influenced the RRs of magnesium more than 10% (age, sex, family history of colorectal cancer, body mass index (BMI), physical activity, energy-adjusted intakes of fat, fiber, calcium, folate, beta-carotene, vitamins E and B6, alcohol, and energy intake).

## RESULTS

Mean (±s.d.) energy-adjusted magnesium intake was 332 (±58) and 292 (±48) mg day^−1^ among subcohort men and women, respectively. Important sources of magnesium were wholewheat bread, dairy, pulses, coffee, tea, and peanuts/peanut butter. Magnesium supplements were used by only 0.2% of individuals. Baseline characteristics of the subcohort are presented in Supplementary Table. Magnesium intake was weakly inversely associated with colorectal and colon cancer risks in men and women, but nonsignificantly (Table 1). Exclusion of the first 2 years of follow-up yielded similar results. Because men and women showed comparable results, we combined them in analyses stratified by BMI. Table 2 shows that the association with colorectal cancer and its subsites varied by BMI: for those with a BMI⩾25 kg m^−2^, this was inverse (except rectum), with *P*-trend reaching significance for colon, and especially proximal colon cancer. The RRs of proximal colon cancer for increasing quintiles of magnesium were 1.0, 0.69, 0.65, 0.48, and 0.54, respectively (*P*-trend=0.02). For those with BMI <25 kg m^−2^, there was no association with magnesium. Tests for interaction were nonsignificant. Results for men and women separately were essentially similar (data not shown).

## DISCUSSION

An inverse association between magnesium intake and colorectal cancer risk in women was first reported in a Swedish cohort study (Larsson *et al*, 2005). In the Iowa Women's Health Study, an inverse association was found only for colon cancer. We found weak inverse associations with risks of colorectal and colon cancer in men and women, which were generally nonsignificant. In both sexes, the inverse association was most evident for proximal colon cancer risk. When we stratified by BMI level, the inverse association was observed only in those with BMI ⩾25 kg m^−2^. As overweight is related to decreased insulin sensitivity (Fung *et al*, 2003), this may suggest that magnesium is inversely associated with colorectal cancer risk through improved insulin sensitivity. Recently, magnesium intake was found to be associated with increased levels of adiponectin, which may improve insulin sensitivity (Qi *et al*, 2005); adiponectin was inversely associated with colorectal cancer risk among men (Wei *et al*, 2005).

Strengths of our study include large numbers of cases, scope for comparing the sexes, and the completeness of follow-up. We found weaker inverse associations between colorectal cancer and magnesium intake than in the Sweden (Larsson *et al*, 2005) and, to a lesser extent, Iowa studies (Folsom and Hong, 2006). It may be relevant that reported magnesium intake levels are lower in Sweden than in the Netherlands: median intakes in lowest and highest quintiles were 198 and 268 mg day^−1^ (Larsson *et al*, 2005), and 236 and 349 mg day^−1^ in Dutch women, respectively. Magnesium intake of up to 325 mg day^−1^ was recently found to be associated with insulin sensitivity, and intakes above this level might not provide further benefits; sex-specific data were not presented (Ma *et al*, 2006). We observed no further decrease in risk in our subsite-specific analyses (Table 2) in quintile 5 (>350 mg day^−1^; median 375) compared to quintile 4 (321–350 mg day^−1^; median 313), which is in line with the threshold finding. The magnesium intake in Iowa women (Folsom and Hong, 2006) was comparable to our study, but Iowa women were generally heavier (Folsom and Hong, 2006) than Dutch women, which could explain the different findings given the modification by BMI.

In conclusion, our results provide no clear support for an overall protective effect of magnesium on colorectal cancer in men or women, but are compatible with an impact in the subgroup of overweight subjects, possibly through reduced insulin resistance. Further studies are needed to elucidate this relationship.

## Figures and Tables

**Table 1:** Relative rates (RRs) of colorectal cancer according to energy-adjusted magnesium intake, Netherlands Cohort Study 1986–1999

	**Quintiles of energy-adjusted magnesium intake (mg day^−1^)**					
	**Q1**	**Q2**	**Q3**	**Q4**	**Q5**	***P*-trend**
*Men*						
Quintile cutoffs (mg day^−1^)	<286	286–316	317–341	342–373	>373	
Median (mg day^−1^)	264	303	329	356	401	
Person-years in subcohort	4761	4823	4919	4836	4757	
						
*Colorectal cancer*						
Cases	275	281	297	264	263	
Age-adjusted RR^a^	1.0	1.00 (0.80–1.25)	1.04 (0.83–1.29)	0.95 (0.76–1.18)	0.96 (0.77–1.20)	0.57
Multivariate RR^b^	1.0	0.96 (0.75–1.22)	0.96 (0.74–1.26)	0.87 (0.64–1.17)	0.91 (0.62–1.35)	0.50
						
*Colon cancer*						
Cases	192	180	185	167	159	
Age-adjusted RR^a^	1.0	0.92 (0.71–1.18)	0.92 (0.72–1.18)	0.86 (0.67–1.10)	0.83 (0.64–1.07)	0.13
Multivariate RR^b^	1.0	0.89 (0.67–1.17)	0.87 (0.64–1.19)	0.82 (0.59–1.15)	0.85 (0.54–1.33)	0.41
						
*Proximal colon cancer*						
Cases	77	81	86	73	64	
Age-adjusted RR^a^	1.0	1.03 (0.73–1.45)	1.07 (0.76–1.50)	0.94 (0.66–1.33)	0.83 (0.58–1.20)	0.27
Multivariate RR^b^	1.0	0.95 (0.65–1.38)	0.95 (0.63–1.43)	0.81 (0.51–1.28)	0.73 (0.39–1.36)	0.28
						
*Distal colon cancer*						
Cases	103	90	95	90	85	
Age-adjusted RR^a^	1.0	0.85 (0.62–1.17)	0.88 (0.65–1.21)	0.86 (0.63–1.18)	0.82 (0.60–1.14)	0.30
Multivariate RR^b^	1.0	0.84 (0.59–1.19)	0.86 (0.58–1.27)	0.87 (0.57–1.31)	0.94 (0.53–1.64)	0.85
						
*Rectum cancer* c						
Cases	83	101	112	97	104	
Age-adjusted RR^a^	1.0	1.19 (0.86–1.65)	1.29 (0.94–1.78)	1.15 (0.83–1.59)	1.25 (0.91–1.73)	0.27
Multivariate RR^b^	1.0	1.12 (0.79–1.59)	1.18 (0.80–1.73)	0.99 (0.63–1.55)	1.07 (0.61–1.89)	0.94
						
*Women*						
Quintile cutoffs (mg day^−1^)	<256	256–279	280–300	301–326	>326	
Median (mg day^-1^)	236	269	289	313	349	
Person-years in subcohort	4902	5152	5157	5014	5258	
						
*Colorectal cancer*						
Cases	217	185	172	186	188	
Age-adjusted RR^a^	1.0	0.84 (0.66–1.07)	0.79 (0.62–1.01)	0.88 (0.69–1.12)	0.88 (0.69–1.12)	0.42
Multivariate RR^b^	1.0	0.83 (0.63–1.08)	0.78 (0.58–1.06)	0.89 (0.63–1.24)	0.89 (0.59–1.35)	0.77
						
*Colon cancer*						
Cases	159	136	127	135	138	
Age-adjusted RR^a^	1.0	0.94 (0.64–1.10)	0.80 (0.61–1.05)	0.87 (0.66–1.14)	0.88 (0.67–1.15)	0.45
Multivariate RR^b^	1.0	0.83 (0.62–1.12)	0.79 (0.57–1.11)	0.89 (0.61–1.29)	0.89 (0.56–1.40)	0.77
						
*Proximal colon cancer*						
Cases	95	70	64	70	84	
Age-adjusted RR^a^	1.0	0.73 (0.52–1.03)	0.68 (0.48–0.97)	0.77 (0.54–1.08)	0.92 (0.66–1.28)	0.70
Multivariate RR^b^	1.0	0.71 (0.49–1.03)	0.66 (0.44–1.01)	0.75 (0.47–1.20)	0.86 (0.49–1.52)	0.69
						
*Distal colon cancer*						
Cases	58	61	60	59	50	
Age-adjusted RR^a^	1.0	1.02 (0.69–1.50)	1.01 (0.69–1.49)	1.02 (0.69–1.51)	0.84 (0.56–1.26)	0.46
Multivariate RR^b^	1.0	1.03 (0.67–1.59)	1.03 (0.63–1.67)	1.09 (0.64–1.88)	0.93 (0.47–1.84)	0.98
						
*Rectum cancer* c						
Cases	58	49	45	51	50	
Age-adjusted RR^a^	1.0	0.83 (0.55–1.25)	0.78 (0.51–1.18)	0.90 (0.60–1.35)	0.87 (0.58–1.31)	0.67
Multivariate RR^b^	1.0	0.81 (0.52–1.25)	0.76 (0.46–1.25)	0.89 (0.51–1.55)	0.91 (0.46–1.79)	0.90

a Data presented as RR (95% confidence interval).

b The model included age, family history of colorectal cancer, BMI, physical activity, energy-adjusted intakes of fat, fibre, calcium, folate, beta-carotene, vitamin E, vitamin B6, alcohol, and energy intake.

c Includes rectosigmoid.

**Table 2:** Relative rates (RRs) of colorectal cancer according to magnesium intake and BMI in men and women combined, Netherlands Cohort Study 1986–1999

	**Quintiles of energy-adjusted magnesium intake (mg day^−1^)**					
	**Q1**	**Q2**	**Q3**	**Q4**	**Q5**	***P*-trend**
Quintile cutoffs (mg day^−1^)	<270	271–298	299–320	321–350	>350	
Median (mg day^−1^)	248	286	309	335	375	
Person-years in subcohort	9707	9939	9956	9902	10 077	
						
*Colorectal cancer*						
Cases	522	472	451	433	450	
Multivariate RR^a^	1.0	0.91 (0.76–1.09)	0.89 (0.73–1.08)	0.88 (0.70–1.10)	0.93 (0.70–1.23)	0.56
						
*BMI <25 kg m^−2^*						
Cases	257	250	217	229	235	
Multivariate RR^a^	1.0	1.05 (0.82–1.35)	0.99 (0.75–1.31)	1.14 (0.83–1.57)	1.11 (0.75–1.64)	0.51
						
*BMI ⩾25 kg m^−2^*						
Cases	265	222	234	204	215	
Multivariate RR^a^	1.0	0.77 (0.59–1.01)	0.79 (0.59–1.05)	0.67 (0.48–0.93)	0.77 (0.50–1.18)	0.14
						
*Colon cancer*						
Cases	365	327	298	290	298	
Multivariate RR^a^	1.0	0.89 (0.73–1.09)	0.83 (0.67–1.05)	0.85 (0.66–1.10)	0.91 (0.66–1.25)	0.48
						
*BMI <25 kg m^−2^*						
Cases	172	170	141	153	160	
Multivariate RR^a^	1.0	1.09 (0.82–1.44)	0.99 (0.72–1.37)	1.20 (0.84–1.72)	1.22 (0.79–1.91)	0.34
						
*BMI ⩾25 kg m^-2^*						
Cases	193	157	157	137	138	
Multivariate RR^a^	1.0	0.72 (0.53–0.96)	0.69 (0.50–0.95)	0.60 (0.42–0.87)	0.67 (0.41–1.08)	0.05
						
*Proximal colon cancer*						
Cases	169	167	145	134	149	
Multivariate RR^a^	1.0	0.91 (0.70–1.18)	0.80 (0.59–1.07)	0.75 (0.54–1.04)	0.82 (0.54–1.25)	0.18
						
*BMI <25 kg m^−2^*						
Cases	78	87	64	71	80	
Multivariate RR^a^	1.0	1.19 (0.82–1.72)	0.96 (0.62–1.47)	1.13 (0.71–1.81)	1.25 (0.70–2.22)	0.64
						
*BMI ⩾25 kg m^−2^*						
Cases	91	80	81	63	69	
Multivariate RR^a^	1.0	0.69 (0.47–1.01)	0.65 (0.43–0.98)	0.48 (0.30–0.78)	0.54 (0.29–1.00)	0.02
						
*Distal colon cancer*						
Cases	176	149	144	147	135	
Multivariate RR^a^	1.0	0.89 (0.68–1.17)	0.90 (0.67–1.22)	1.00 (0.72–1.39)	0.99 (0.64–1.53)	0.81
						
*BMI <25 kg m^−2^*						
Cases	81	77	70	76	71	
Multivariate RR^a^	1.0	1.10 (0.75–1.60)	1.11 (0.72–1.71)	1.39 (0.87–2.24)	1.29 (0.71–2.36)	0.26
						
*BMI ⩾25 kg m^−2^*						
Cases	95	72	74	71	64	
Multivariate RR^a^	1.0	0.72 (0.49–1.07)	0.74 (0.48–1.13)	0.74 (0.46–1.18)	0.77 (0.40–1.49)	0.49
						
*Rectum cancer^b^*						
Cases	157	145	153	143	152	
Multivariate RR^a^	1.0	0.95 (0.72–1.25)	1.02 (0.75–1.38)	0.95 (0.67–1.35)	0.99 (0.64–1.52)	0.98
						
*BMI <25 kg m^−2^*						
Cases	85	80	76	76	75	
Multivariate RR^a^	1.0	0.98 (0.67–1.43)	0.99 (0.65–1.52)	1.05 (0.64–1.72)	0.92 (0.50–1.71)	0.95
						
*BMI ⩾25 kg m^−2^*						
Cases	72	65	77	67	77	
Multivariate RR^a^	1.0	0.91 (0.60–1.39)	1.07 (0.69–1.67)	0.85 (0.50–1.44)	1.06 (0.54–2.05)	0.98

a The model included age, sex, family history of colorectal cancer, BMI, physical activity, energy-adjusted intakes of fat, fibre, calcium, folate, beta-carotene, vitamin E, vitamin B6, alcohol, and energy intake.

b Includes rectosigmoid.
