# Supplementary material for: Magnesium intake and colorectal cancer risk in the Netherlands Cohort Study
Source: Br J Cancer. 2007 Feb 6;96(3):510–3. doi: 10.1038/sj.bjc.6603577 (PMC2360034; doi:10.1038/sj.bjc.6603577)
Supplement: Supplementary table [file 6603577x1.doc]

WEB (internet) Table. Baseline characteristics of subcohort members (n= 4438) according to magnesium intake in the Netherlands Cohort Study, 1986.

| Characteristic | Quintiles of energy-adjusted magnesium intake (mg/d) | | | | | | | | | | |
| --- | --- | --- | --- | --- | --- | --- | --- | --- | --- | --- | --- |
|  | Men | | | | |  | Women | | | | |
|  | 1 | 2 | 3 | 4 | 5 |  | 1 | 2 | 3 | 4 | 5 |
|  | < 286 | 286-316 | 317-341 | 342-373 | > 373 |  | < 256 | 256-279 | 280-300 | 301-326 | > 326 |
| No. of participants | 439 | 437 | 439 | 438 | 438 |  | 449 | 449 | 450 | 450 | 449 |
| Age at baseline (yrs), mean (SD) | 61.4 (4.2) | 61.4 (4.3) | 61.5 (4.2) | 61.3 (4.2) | 61.1 (4.2) |  | 62.1 (4.4) | 61.4 (4.3) | 61.3 (4.3) | 61.4 (4.2) | 60.8 (4.1) |
| Family history (yes), % | 3.6 | 5.7 | 5.9 | 5.9 | 4.8 |  | 5.1 | 7.8 | 5.1 | 6.2 | 4.7 |
| BMI (kg/m^2^), mean (SD) | 25.1 (2.8) | 25.0 (2.3) | 24.9 (2.7) | 25.2 (2.6) | 24.6 (2.4) |  | 24.7 (3.8) | 24.9 (3.6) | 25.2 (3.6) | 25.3 (3.3) | 25.2 (3.6) |
| Physical activity (nonoccup.) |  |  |  |  |  |  |  |  |  |  |  |
| < 30 min/day | 24.1 | 18.9 | 18.2 | 15.4 | 14.8 |  | 30.8 | 28.4 | 22.7 | 21.8 | 21.0 |
| 30-60 min/day | 32.0 | 30.6 | 32.0 | 29.7 | 31.6 |  | 31.7 | 31.4 | 34.0 | 30.6 | 28.2 |
| 60-90 min/day | 14.5 | 19.4 | 20.7 | 20.5 | 18.5 |  | 20.4 | 21.4 | 22.3 | 26.3 | 21.8 |
| > 90 min/day | 29.4 | 31.1 | 29.0 | 34.5 | 35.1 |  | 17.2 | 18.7 | 21.0 | 21.3 | 29.0 |
| Educational level, % |  |  |  |  |  |  |  |  |  |  |  |
| Low | 48.3 | 47.3 | 43.2 | 47.1 | 41.6 |  | 59.1 | 60.6 | 55.1 | 54.8 | 52.1 |
| Medium | 36.5 | 35.0 | 35.1 | 33.6 | 36.8 |  | 32.3 | 20.3 | 36.7 | 35.8 | 36.5 |
| High | 15.3 | 16.9 | 21.0 | 18.5 | 21.5 |  | 7.8 | 8.0 | 8.0 | 9.3 | 10.7 |
| Smoking status, % |  |  |  |  |  |  |  |  |  |  |  |
| Never | 10.7 | 14.0 | 12.8 | 11.2 | 14.8 |  | 57.7 | 58.8 | 63.8 | 55.8 | 55.9 |
| Ex | 43.5 | 45.1 | 53.5 | 56.2 | 59.6 |  | 17.4 | 18.0 | 20.2 | 21.1 | 26.3 |
| Current | 45.8 | 41.0 | 33.7 | 32.6 | 25.6 |  | 24.9 | 23.2 | 16.0 | 23.1 | 17.8 |
| Energy intake (kcal/d),mean (SD) | 2213 (506) | 2137 (511) | 2124 (472) | 2112 (514) | 2242 (535) |  | 1723 (407) | 1700 (430) | 1601 (356) | 1673 (377) | 1731 (400) |
| Fat (g/d), mean (SD)^a^ | 95.3 (14.9) | 95.3 (14.4) | 95.3 (12.7) | 93.7 (13.2) | 90.1 (15.3) |  | 76.4 (12.0) | 75.6 (9.7) | 74.9 (8.7) | 73.6 (9.0) | 69.3 (10.3) |
| Fibre (g/d), mean (SD)^a^ | 21.4 (4.0) | 25.3 (4.3) | 28.3 (4.1) | 31.3 (4.7) | 37.0 (7.0) |  | 19.5 (3.8) | 23.1 (3.8) | 25.1 (3.7) | 27.4 (3.9) | 31.3 (5.3) |
| Calcium (mg/d), mean (SD)^a^ | 775 (237) | 890 (246) | 946 (271) | 1010 (256) | 1130 (334) |  | 716 (207) | 816 (212) | 904 (213) | 957 (233) | 1109 (283) |
| Folate (μg/d), mean (SD)^a^ | 252.2 (42.0) | 280.4 (44.1) | 298.5 (45.3) | 323.5 (53.6) | 361.3 (73.7) |  | 219.8 (40.3) | 251.0 (39.9) | 277.2 (46.2) | 294.8 (44.6) | 338.9 (63.3) |
| Beta-carotene (g/d), mean (SD)^a^ | 0.3 (0.2) | 0.4 (0.2) | 0.4 (0.2) | 0.4 (0.2) | 0.5 (0.3) |  | 0.3 (0.2) | 0.4 (0.2) | 0.4 (0.2) | 0.5 (0.2) | 0.6 (0.3) |
| Vitamin E (mg/d), mean (SD)^a^ | 13.5 (5.5) | 14.3 (5.6) | 14.9 (5.3) | 15.1 (5.4) | 15.7 (6.1) |  | 11.6 (4.4) | 12.0 (4.5) | 12.0 (4.2) | 12.5 (4.5) | 12.3 (4.6) |
| Vitamin B6 (mg/d), mean (SD)^a^ | 1.3 (0.2) | 1.5 (0.2) | 1.6 (0.2) | 1.6 (0.2) | 1.7 (0.3) |  | 1.1 (0.2) | 1.3 (0.2) | 1.4 (0.2) | 1.4 (0.2) | 1.5 (0.2) |
| Alcohol (g/d), mean (SD) | 19.9 (21.4) | 16.5 (18.4) | 13.0 (13.0) | 13.4 (14.3) | 12.1 (14.4) |  | 6.6 (11.9) | 6.2 (9.6) | 5.8 (9.2) | 5.9 (8.7) | 4.9 (7.8) |
| Heme (mg/d), mean (SD)^a^ | 1.1 (0.4) | 1.2 (0.4) | 1.2 (0.5) | 1.2 (0.5) | 1.2 (0.5) |  | 0.9 (0.4) | 1.0 (0.4) | 1.0 (0.4) | 1.0 (0.5) | 1.0 (0.6) |
| Chlorofyll (mg/d), mean (SD)^a^ | 43.0 (24.3) | 49.1 (24.2) | 53.0 (25.6) | 55.8 (30.0) | 64.0 (39.3) |  | 40.0 (23.7) | 46.9 (24.1) | 53.4 (27.3) | 56.7 (29.3) | 65.8 (35.0) |

^a^ Energy-adjusted nutrient variables.
